# Interdisciplinary Tensions When Developing Digital Interventions Supporting Individuals With ADHD

**DOI:** 10.3389/fdgth.2022.876039

**Published:** 2022-05-12

**Authors:** Franceli L. Cibrian, Elissa Monteiro, Sabrina E. B. Schuck, Michele Nelson, Gillian R. Hayes, Kimberley D. Lakes

**Affiliations:** ^1^Fowler School of Engineering, Chapman University, Orange, CA, United States; ^2^Graduate School of Education, University of California, Riverside, Riverside, CA, United States; ^3^Pediatrics Department, University of California, Irvine, Irvine, CA, United States; ^4^Department of Psychiatry and Neuroscience, University of California, Riverside, Riverside, CA, United States; ^5^Informatics Department, University of California, Irvine, Irvine, CA, United States

**Keywords:** Digital Health Intervention, ADHD, mental health, human-computer interaction, development

## Introduction

Attention Deficit/Hyperactivity Disorder (ADHD) is the most prevalent childhood psychiatric condition, with a worldwide prevalence estimate between 5% and 7.2% (1, 2), affecting nearly 9.4% of children among 2–17 years old in the United States (3). Individuals with ADHD display symptoms of inattention (e.g., they are most notably easily distracted and have trouble sustaining attention for a prolonged amount of time), hyperactive/impulsive behaviors, and difficulty regulating their bodies and emotions (4). Behavioral interventions are promising as approaches to improving the control of attention and impulsivity (5), especially when using technological interventions, which have shown promising results in supporting children and adults with ADHD [see recent reviews (6–8)].

Our collaborative team of experts in psychology, psychiatry, computer science, and human-computer interaction (HCI) recently published a book on Digital Health Interventions (DHI) for individuals with Attention Deficit Hyperactivity Disorder (ADHD) and related difficulties (6), and two review papers (7, 8) in which we focused on two domains of scientific inquiry: design and computing—which includes computer and information sciences, HCI, and related fields—and clinical, which includes medical and psychological fields.

Our analysis observed tensions between these two fields around the research project lifecycles, requirements and design methods, implementation, evaluation methods, and measurement. Blandford et al. (9) described these contrasts in practice between HCI and health^1^, noting how tensions complicate translation across fields. These difficulties ultimately impact the potential for adoption by clinicians, patients, and families, resulting in many innovative technologies failing to make a demonstrable impact on health outcomes.

This opinion article aims to draw, from our experiences alongside our recent literature reviews, the interdisciplinary tensions that arise when developing and studying DHI for ADHD to specify recommendations and build a multidisciplinary agenda that will improve the quality and impact of DHI.

## Life Cycles

DHIs for ADHD support diagnosis, assessment, and interventions that target attention, social-emotional skills, self-regulation, motor skills, and academic and vocational skills. Despite a shared interest in these challenges and goals, researchers in the different fields tend to follow different research lifecycles.

HCI researchers typically follow a user-centered design approach that involves identifying user needs, understanding the context of use, and designing digital tools iteratively and collaboratively (10). This approach involves users in all stages of development, from early design to prototyping to full system development and user studies. For example, Sonne et al. (11) conducted a contextual inquiry to design an initial version of MOBERO, a mobile application supporting families that include children with ADHD during morning and bedtime routines. They piloted the tool with two families to gather more requirements and evolve MOBERO functionality. Using their eventual stable DHI, they conducted a deployment study with 13 families to provide evidence of the usability and potential effectiveness of MOBERO (12).

In clinical research, the lifecycle should start with development based on a well-known or evidence-based theory, a hypothesized mechanism of action, which is then followed by pilot testing, randomized controlled trials (RCT), and subsequent implementation studies (13). In a recent mapping review of ADHD and DHI (8), although we were able to identify 51 studies involving DHI for ADHD, only 12 reported RCTs examining DHI outcomes in children or adolescents with ADHD. None of the products developed or studied appeared to have reached the stage of implementation research. One of the RCTs included in our review examined outcomes from Plan-It Commander (14), an internet-based serious game for ADHD children, builds on theories of self-regulation (15), social cognition (16), and learning (17) to teach time management, planning and organizing, and prosocial skills. The game was evaluated in a 20-week RCT with 182 children (aged 8–10 years) with ADHD (14) in which parents and teachers reported improvements in social skills surrounding gameplay, but reports of planning and organizing skills were not significantly different between groups (18).

With a mixed team of clinical and computational scientists, we have embraced an approach that blends user-centered design with clinical research methods. Applying self-regulation theory and evidence-based interventions for ADHD (19), we designed an app to assist parents in supporting the behavioral goals of their child with ADHD and promoting the use of self-regulation strategies in youth with ADHD. To understand the application of theory to practice through DHI, we engage user-center design methods, including co-design with children with ADHD and early user testing and engagement with caregivers (parents and teachers) (20). We are now testing CoolCraig, a mobile and smartwatch application to support a token economy and zone of regulation strategies in a family setting, which resulted from this blend of theoretical and empirical design work (21). We will continue to iterate on this system with the ultimate goal of creating a stable version for an RCT and eventual translation to clinical and educational practice.

## Requirements and Design Methods

Clinical and computational approaches to DHI design for ADHD involve end-users to some degree. However, how their input is considered during the design process varies greatly across projects and fields. Additionally, over time, all the fields in this study appear to be moving towards an ethos of greater inclusion, which can be a difficult shift to norms and culture within and across disciplines.

HCI researchers frequently use field-based and contextual design methods, including ethnographic approaches, for understanding the needs and practices of people with ADHD and related stakeholders. Thus, HCI researchers must develop strategies for engaging individuals with ADHD during these activities, especially when working with children. For example, Fekete and Lucero (22) found that considering children with ADHD's needs, preferences, and desires, in tandem with a structured environment and scaffolds, can motivate them to actively participate in the co-design process of DHI. These kinds of efforts can help HCI researchers as well as user experience professionals, therapists, teachers, and even parents to center the needs and interests of ADHD children in their projects.

In clinical research, designs translate current theories into digital interventions. For example, the first FDA-approved video game for treating children with ADHD (23) was developed following the fundamentals of Neuroracer, a videogame designed to support the multitasking of older adults (24). Neuroracer was adapted into a mobile DHI for children with ADHD that was initially evaluated in a proof of concept study (25) and then in an RCT with 857 patients (26). In this case, the clinicians' selected feasible theories according to their experience that have the most potential to support the clinical outcomes expected.

To balance the inclusion of evidence-based clinical knowledge and the lived experiences of people with ADHD, interdisciplinary teams must develop innovative strategies to ensure attention is paid to all types of expertise. In our research, we balance those approaches by selecting theories from ADHD experts and conducting qualitative research with individuals with ADHD and clinicians, so both have chances to be co-designers of the digital intervention.

## Implementation

In computational fields, fundable and publishable research implementations often must include some innovation in software or hardware. Therefore, it was not surprising that many papers in our past reviews (6) related to proposing algorithms to assess ADHD using different machine learning approaches to classify brain activity (27–40). Similarly, papers often contributed to the scholarly discourse of developing novel prototypes (41–44) in which the efficacy had not been demonstrated. Sometimes with empirical evidence about usability, the prototype itself is considered a contribution in the HCI field (45), including protocols for data privacy and analysis of data gathered from input devices.

On the clinical side, the term implementation is used as the final step “*when a complex intervention (incorporating digital technologies) has been fully tested*” (9). Thus, before the implementation stage, there should be conducted at least efficacy RCTs to fully test the DHI. Most of these types of studies for ADHD either use commercially available devices like mobile phones (46) or personal computers (14, 18) with software or systems that are primarily “off-the-shelf” [e.g., neurofeedback training (47–49)]. Using commercially available systems allows long, complex interventions to test efficacy once usability and safety have already been determined.

As in our design research approach, we take a hybrid approach again by relying on commercially available devices such as iPhones and Apple Watch while including custom-developed applications. In this case, we seek to evaluate the use, adoption, and potential efficacy of novel designs and systems as implemented in so-called “off the shelf” devices.

## Evaluation Methodologies

The “gold standard” to evaluate a DHI in a clinical field is the RCT (50). Such study designs may use control or waitlist conditions with experimental conditions. They involve extensive planning with a finalized digital tool and intervention prior to the trial. Given the stability of design required, RCTs tend to include commercially available applications or devices [e.g., ACTIVATE^TM^ (51–53); RoboMemo (54)], and use standardized assessment with well-established validity and high reliability to assess outcomes. Moreover, the inclusion and exclusion criteria typically require participants to exhibit clinically significant symptoms of ADHD. When studying technological tools focused on diagnosing or assessing ADHD, researchers conduct the diagnosis using standard clinical assessment approaches to determine whether participants meet the diagnostic criteria for the disorder (55, 56) and compare the tool under investigation with these clinical measures. These studies usually require robust and well-diagnosed samples of at least 50 to 100 participants.

In HCI and related fields, a formative evaluation to test the usability, usefulness, acceptability, and user experience can be conducted even with a small number of participants [e.g., (57, 58)] and sometimes “in-the-wild” [e.g., (11, 59, 60)]. The inclusion and exclusion criteria, despite often being as strict as clinical fields, are frequently not well-described in publications [e.g., (61, 62)]. Formal diagnostic assessment is often not conducted or required for participation in these studies.

The differences in approaches draw out two clear tensions in DHI research more broadly. In any given research study, a focus on adoption and usability will identify approaches that end users would engage in but may not provide as much evidence for efficacy. A focus on clinically verified approaches will likely mean that the intervention is efficacious, still research participants need to use the tools at a certain dosage to measure that efficacy and will have either been required or incentivized to do so as part of the research study. It is incredibly difficult -if not impossible- in a single research study to measure both whether and how people will use the tool and its effects when used properly.

Currently, in our research, we are conducting a formative evaluation with a small number of participants, using a more HCI approach. However, we also use standardized assessments for pre- and post-evaluations in keeping with clinical research standards and with the aim of moving toward an RCT to examine efficacy.

## Discussion: Recommendations

Our research—design, literature, empirical, and technical— raise important questions about creating DHI that are valid, efficacious, and accepted by end-users, including both people with ADHD and clinicians who might recommend or prescribe them. At the same time, it raises questions about developing innovative technologies that are also stable enough to withstand clinical quality evaluations. As improved software engineering, Artificial Intelligence (AI), and design techniques allow for more rapid prototyping of stable yet innovative tools, we can now conduct clinical studies quickly while still engaging in iterative and interactive design approaches.

Combining empirically based theories of ADHD with contextual design enriches the understanding of requirements. Co-design with people with ADHD and traditional “experts” leads to better and more inclusive design. However, researchers must carefully engage these groups—sometimes separately—to ensure that all the voices are heard, and a variety of views are taken into account.

Although “implementation” has a very different meaning in both fields (implement the design solution vs. implement the DHI in the long term), the better an HCI implementation is done, the more likely it is that clinicians will take up the solution in practice.

As an emergent multidisciplinary field, researchers working on DHI for ADHD should commit to describing participants (samples) similarly, providing details about software and hardware implementation and the context of use. Likewise, researchers must commit to creating usable and appealing DHI as equally important goals to creating clinically efficacious, evidence-based tools. Accomplishing both requires a commitment, upfront, to the resources, time, and effort required (63). Publication standards must also allow greater flexibility in multidisciplinary approaches, such that researchers can engage communities around both initial design probes and longer and larger studies leading to an RCT. Indeed, a spectrum of approaches must be applauded, not simply allowed. As technologies change rapidly and family contexts develop as children grow, this flexibility is essential when considering DHI for ADHD.

Ultimately, literature searches, publication standards, and dissemination norms must allow HCI researchers to learn more about clinical theory and clinicians and clinical researchers' practices to engage with and appreciate iterative design approaches of HCI. Interdisciplinary and diverse teams are needed to create innovative DHIs that translate ideas and prototypes into commercially available products. While we have focused on the fields from which our interdisciplinary team originates in this article, we recognize that a broad interdisciplinary approach across more fields would be ideal for truly innovative but also saleable and sustainable research tested approaches to ADHD more broadly. Moreover, those teams need financial support to conduct pilot testing at the early stages of technology development, but the cost increases once they conduct clinical trials. Consideration must also be made for the challenge between the time it takes for technology to be updated (or become obsolete) and the time required for interdisciplinary teams to obtain sufficient funding for testing. Once teams receive sufficient grant support, substantial modifications to the study design may already be required. Also, obtaining support for interdisciplinary research poses its own challenges; at least in the United States, there is an institution focused on funding “clinical research” (i.e., National Institutes of Health: NIH) and another focused on investing in non-medical fields (i.e., National Science Foundation: NSF). In recent years, efforts to bridge this divide have been developed and used to a certain degree (e.g., calls from NIH for DHIs and collaborative grant opportunities from the NIH and NSF). However, further systemic changes are needed to develop DHIs not only for ADHD but for other groups that could benefit from DHIs.

## Author Contributions

FC, GH, and KL contributed to the conception of the paper. FC and EM organized the information and wrote the first draft of the manuscript. FC, GH, SS, MN, and KL wrote sections for the manuscript. All authors contributed to manuscript revision, read, and approved the submitted version.

## Funding

Research reported in this paper was supported by AHRQ under award number 1R21HS026058, a Jacobs Foundation Advanced Research Fellowship, and the Jacobs Foundation CERES network.

## Author Disclaimer

The content is solely the responsibility of the authors and does not necessarily represent the official views of the funding organizations.

## Conflict of Interest

The authors declare that the research was conducted in the absence of any commercial or financial relationships that could be construed as a potential conflict of interest.

## Publisher's Note

All claims expressed in this article are solely those of the authors and do not necessarily represent those of their affiliated organizations, or those of the publisher, the editors and the reviewers. Any product that may be evaluated in this article, or claim that may be made by its manufacturer, is not guaranteed or endorsed by the publisher.

## Footnotes

^1^Blanford et al. (9) proposed seven areas, but in this paper, we will provide our opinion of four of them related with the lifecycle.
